# Contributions to Experimental Physiology

**Published:** 1852-08

**Authors:** 


### EDITORIAL.

"*Contributions to Experimental Physiology.*"—By BENNET DOWLER, M. D., Corresponding Member of the Academy of Natural Sciences of Philadelphia; Fellow of the Medico-Chirurgical College of the same city, &c., &c.

DR. DOWLER is one of the few men of the present day who have the independence to think for themselves, without reference to the opinions of others, and the disposition to labor for the development of truth, regardless of its effects on popular theories. His new and startling propositions, drawn from experiments made upon the great saurian of the Mississippi, have surprised the profession both in this country and in Europe, while the confidence with which he has expressed, as well as ability with which he has maintained his views, has won for him the respect of scientific men. He has met with opposition, but has not stopped to battle for words; at the shrine of truth he has continued to sacrifice, recording faithfully the answer to his offering.

We have in the work before us the details of two vivisections made upon the alligator, in the presence of several professional gentleman. In the first, the trachea was tied firmly in the middle of the neck, and in about thirty minutes the animal appeared to be completely dead. The dissections had already been commenced when suddenly, to the astonishment of the operator and witnesses, it recovered its feet and assumed an attitude of defence. After ten or fifteen minutes it was recaptured, its spinal cord divided in the cervical and afterwards in the dorsal region; the viscera removed from the body; the nerves of the limbs exposed and finally cut away; and lastly the spinal cord itself completely destroyed by inserting a punch from the dorsal division downwards to the caudal extremity, and upwards to the cervical division.

"From the first to the last division of the cord—from the resuscitation to the close of the experiments, the threefold division of the body made by the two sections of the cord, displayed in all three of its parts, both sensation, volition, and accurately adapted muscular motion. The eyes winked or nictated. The head, towards the close of the experiments, attempted to bite Dr. Reynolds.

The lumbar and caudal division gave the most unequivocal indications of pain, contrivance, and adaptive action. Thus the animal, on being suspended by the neck, so that the legs might hang down, was pricked with a scapel in the groin, whereupon it raised one hind leg, (the other had been amputated) it carried the foot (the law of gravity opposing) instantly and accurately to the exact spot where the injury was inflicted, pushing strongly against the knife, slightly wounding its ancle in the attempt to remove the pain-giving instrument—a feat requiring extreme flexion—a complete doubling of the leg upon the thigh. Now this flexion, and several others performed near the close of the experiments by the remaining hind leg and by the fore legs, took place not only after two divisions of the cord, but after the removal of the individual nerves in the limbs themselves, and after the removal of the viscera and sympathetic nerves, plexuses, and ganglions. These motions indicated sensation and volition, as truly as those of the undivided normal animal. In the divided, eviscerated animal, with its limbs *deprived of its nerves*, clear indications of pain and combined motions took place, when, at the close of the experiments, the divided ends of the cord were touched. Thus, when the dorso-caudal part of the cord was irritated, the hind leg was strongly directed to that place. This it repeated until the entire cord was gradually destroyed by a punch reaching down to the tail. The same phenomena occurred when the punch pressed or disentegrated the cord, from the last, or dorsal division, upward toward the head or cervical division.

"In the dissection of the nerves of the limbs during and after the apparent death—after the first and second divisions of the cord, and after amputation, a certain peculiar kind of muscular twitching, particularly in the fingers and toes, took place from compressing or injuring the nerve-cords (as I have formerly described); some slight compression produced many twitches—a strong disorganizing one, or a section gave one or two only, after which compression in the same place produced no motion whatever. The motion could always be reproduced whenever a new portion of the nerve was selected, provided it was invariably upon the distal side of the disorganization or section. The proximal end, that is, the end connected with the cord, when thus treated, was not succeeded by any motion, while that of the distal portion rather augmented as the irritation approached the extreme distribution of the nerves upon the fingers and toes. When the nerves were uninjured in any way, these twitchings were no greater than after the section of the nerve or the amputation of the limb. These twitchings seem totally void of volition or adaptation, being equally independent of the cord and of the proximal end of the nerves."

The vivisection No. 2 was made with reference to a previously prepared programme which, with the answers to the interrogatories furnished by the experiments, was as follows:

*Programme of Vivisection.*

1. Divide the cervical cord: Will each division continue to manifest sensation and voluntary motion? Will each division act in concert or simultaneously on irritating either at, near, and remote from the line of division. [Ans. Yes.]

2. Divide the lower dorso-lumbar cord: Will all three parts afterwards manifest sensation and voluntary motion? Will two or three act simultaneously for a common end, where the middle or extremities are injured? [Ans. Yes.]

3. Dissect the branchial plexus of nerves from a fore-leg: Will voluntary motion and sensation still continue to manifest themselves on irritating the axilla, and the dorsal and the cervical ends of the cord? Will the bare dissected muscles, if pinched or pricked, contract? [Ans. Yes.]

4. Destroy the principal trunks of the sympathetic: Will this dissection excite or destroy sensation and voluntary motion? [Ans. Dissection excites these, but the destruction of the ganglions and plexuses of the sympathetic does not appear to hasten their extinction. See Dr. Dalton's very interesting experiments accompanying this paper. Dr. Dalton must have cut away the chief part of the splanchnic nerve, together with the solar, cœlic, hepatic, gastric coronary, splenic, mesenteric, renal, spermatic, aortic, and cardiac plexuses, as well as numerous ganglions.]

5. Dissect the spinal roots of that part of the cord which gives off the nerves to the hind legs: Will irritation of the posterior or supposed sensory root not be wholly devoid of muscular action? [Ans. No.] Will irritation of the anterior or so-called motor root, afford sensation or other phenomena like those of the posterior root? [Ans. Yes.]

6. After destroying the spinal roots, remove the corresponding portion of the cord: What effect will pricking and pinching at the groin produce? [Ans. Voluntary motion, if I remember rightly; but in former cases this most certainly took place.]

7. Dissect, pinch, tie, cut, and disorganize the ischiatic or sciatic plexus, and trace the sciatic, the popliteal, anterior tibial, and peroneal nerves. Prick and compress the isolated muscles: Will they not twitch equally with and without nerves?—with and without connection with the cord? [Ans. Yes.]

8. Amputate a limb: Dissect away its nerves, prick and compress its muscles, will not the contractions be equally active as before? [Ans. Yes.]

In explanation of the phenomena observed, Dr. Dowler suggests that the several segments of the cord, which appears to be essential to sensation and self determined motion, may be connected by filaments passing along the roots of the spinal nerves, and perhaps communicating with the great sympathetic system. This connection may not be visible to the naked eye, and can only be interrupted by removing all the organic ganglions and plexuses; a thing scarcely possible to be accomplished during the period of a vivisection, hence the co-ordinate movements of different parts excited by sensations and designed, as was manifested during the experiment, for a specific purpose.

The objections urged by critics against these experiments as illustrations of human physiology are met by Dr. D. with tact and ability. In concluding his remarks he has the following:

But sciolists may exclaim—what has all this to do with human physiology? Are alligators like men? Not exactly. I have acknowledged the difference upon former occasions, perhaps to an unwarrantable extent.* I have a better right to the benefit of the objection than these gentlemen, because they profess to follow the celebrated Carpenter, Todd, Bowman, Hall, and others, who, in their latest and most elaborate works, insist that the cold-blooded animals are the most reliable ones for physiological experiments: Messrs. Todd and Bowman, in their most excellent work, now in the course of publication, namely, "Physiological Anatomy and Physiology of Man," say "*That the nervous force endures much longer in the cold-blooded animals*"—"*On this account the cold-blooded animals must be selected for exhibiting the phenomena*"—a proposition which Prof. Carpenter iterates and reiterates, particularly in his learned work, "Physiology, General and Comparative," just republished in this country. Now, if dissenting gentlemen were more consistent in their objections, their logic would be none the worse for their philosophical reputation. If they can believe that European frogs and turtles illustrate human physiology, why should they reject the *Crocodilus Mississippiensis*, albeit the wisest, biggest, and most perfect beast of the cold-blooded class, as the physiologists of the old world have the justice to acknowledge? Does the original curse against reptilians apply to the alligator only, so as to render it unfit for physio-

* A foreign critic, in 1847, who intended to do me all possible damage, speaks on this wise: "Can any one, we ask, entertain a doubt that, the conditions being the same, the consequences would be the same in man, with a spinal centre constructed upon essentially similar principles to that of reptiles and animals? If such kind of evidence be rejected, physiology must return to its very infancy, for, with few exceptions, little or nothing can be learnt, strange as it may sound to some ears, of human physiology from observations restricted exclusively to man." A Dutch Governor tried to please all, but finding that impossible, determined to hear only one side of every case, as he found that hearing both sides not only confused his mind, but gave the trouble of changing the first opinion and forming a new one!

logical experiments? It is evident that *it* was not the reptile which deceived Eve; for it does *not* "go upon its belly all the days of its life." It walks on four legs! The curse that clings to it is that of being a native American and not a European! Verily an Alligator "hath no honor in his own country," although anatomically and physiologically he combines to a greater extent than any other single animal the essential types characterizing the vertebrata and articulata, approaching birds and mammals on the one side, and rising above the fishes, worms and mollusks on the other. Can the resistance-men prove that crocodilian digestion, absorption, sanguification, nutrition, secretion, circulation, volition, motion, hearing, seeing, and feeling, are altogether different in nature, not simply in degree, from those functions in man? Take the strongest example of contrast, namely, the tenacity of life in the saurian: because life persists longer in the latter than in the former, after extensive injuries, does it follow that the vital actions of the one are essentially different in nature as well as in degree from the other?

Can any unprejudiced and enlightened mind upon a careful review of the above-mentioned experiments, and many others which I have made and published, reconcile them with the following statements?—statements founded almost entirely upon Sir C. Bell's experiments—which experiments Bell said were but very few, and even these few he had no confidence in, as he emphatically declares! Todd and Bowman say: "The anterior root of each spinal nerve is motor—the posterior sensitive. The irritation of the latter gives rise to no muscular action. Comparative anatomy confirms this conclusion among all classes of vertebrate animals. The origin of a double root denotes a double function. The union of the encephalon with the spinal cord is necessary for voluntary motion and for sensation."

We are glad that the doctor continues to work, even though he has no doctrines to establish, no theories to sustain. The truths which he is developing may be the materials out of which others shall construct systems. It may be his to furnish and polish and inscribe the blocks which other hands shall place in the column which is being built of the results of human effort, yet, as each block in our own national monument does honor to the State that gave it, so each truth in the massive structure that human intellect is rearing will be to its discoverer a "monumentum ære perennius."

